# Structural and functional basis for pan-CoV fusion inhibitors against SARS-CoV-2 and its variants with preclinical evaluation

**DOI:** 10.1038/s41392-021-00712-2

**Published:** 2021-07-29

**Authors:** Shuai Xia, Qiaoshuai Lan, Yun Zhu, Chao Wang, Wei Xu, Yutang Li, Lijue Wang, Fanke Jiao, Jie Zhou, Chen Hua, Qian Wang, Xia Cai, Yang Wu, Jie Gao, Huan Liu, Ge Sun, Jan Münch, Frank Kirchhoff, Zhenghong Yuan, Youhua Xie, Fei Sun, Shibo Jiang, Lu Lu

**Affiliations:** 1 Key Laboratory of Medical Molecular Virology (MOE/NHC/CAMS), School of Basic Medical Sciences and Biosafety Level 3 Laboratory, Shanghai Institute of Infectious Disease and Biosecurity, Fudan University, Shanghai, China; 2 National Key Laboratory of Biomacromolecules, CAS Center for Excellence in Biomacromolecules, Institute of Biophysics, Chinese Academy of Sciences, Beijing, China; 3 State Key Laboratory of Toxicology and Medical Countermeasures, Beijing Institute of Pharmacology and Toxicology, Beijing, China; 4 China Institute for Radiation Protection, Taiyuan, Shanxi China; 5 Institute of Molecular Virology, Ulm University Medical Center, Ulm, Germany; 6 Bioland Laboratory (Guangzhou Regenerative Medicine and Health Guangdong Laboratory), Guangzhou, Guangdong China

**Keywords:** Structural biology, Infectious diseases

## Abstract

The COVID-19 pandemic poses a global threat to public health and economy. The continuously emerging SARS-CoV-2 variants present a major challenge to the development of antiviral agents and vaccines. In this study, we identified that EK1 and cholesterol-coupled derivative of EK1, EK1C4, as pan-CoV fusion inhibitors, exhibit potent antiviral activity against SARS-CoV-2 infection in both lung- and intestine-derived cell lines (Calu-3 and Caco2, respectively). They are also effective against infection of pseudotyped SARS-CoV-2 variants B.1.1.7 (Alpha) and B.1.1.248 (Gamma) as well as those with mutations in S protein, including N417T, E484K, N501Y, and D614G, which are common in South African and Brazilian variants. Crystal structure revealed that EK1 targets the HR1 domain in the SARS-CoV-2 S protein to block virus-cell fusion and provide mechanistic insights into its broad and effective antiviral activity. Nasal administration of EK1 peptides to hACE2 transgenic mice significantly reduced viral titers in lung and intestinal tissues. EK1 showed good safety profiles in various animal models, supporting further clinical development of EK1-based pan-CoV fusion inhibitors against SARS-CoV-2 and its variants.

## Introduction

Severe acute respiratory syndrome coronavirus 2 (SARS-CoV-2) is the causative agent of coronavirus disease 2019 (COVID-19). SARS-CoV-2 belongs to the coronaviridae family, β-coronavirus genera, and shows close relationship with SARS-CoV.^1,2^ As of 28 June 2021, SARS-CoV-2 had caused 180,817,269 confirmed cases and 3,923,238 deaths worldwide (https://covid19.who.int/). SARS-CoV-2 transmits mainly through the respiratory pathway and may cause severe respiratory symptoms, such as dyspnea and even pulmonary failure.^3^ Additionally, SARS-CoV-2 RNA has been frequently detected in fecal samples from COVID-19 patients with gastrointestinal symptoms, including abdominal pain, diarrhea, vomiting, anorexia and nausea, suggesting that SARS-CoV-2 may also be transmitted through the fecal–oral pathway,^4,5^ which further increases the difficulty in combating SARS-CoV-2 pandemic.

Additionally, as the global pandemic of SARS-CoV-2 presents different states of infectivity in different countries, many variants have emerged and spread worldwide. For example, the Alpha variant B.1.1.7 emerged in southeastern England in November 2020 and has quickly spread worldwide to become the dominant circulating SARS-CoV-2 variant in many countries.^6^ Recently, B.1.1.248 (Gamma) and B.1.351 (Beta) variants with enhanced transmissibility and/or pathogenicity have also been reported and became dominant in South African variants and Brazilian, respectively.^7,8^ Such SARS-CoV-2 variants have brought a more severe challenge to the prevention and control of the COVID-19 pandemic.

The SARS-CoV-2 spike (S) protein presents on the viral surface and plays a crucial role in mediating viral infection.^9^ S protein can be divided into two subunits, S1 and S2. The S1 subunit contains the receptor binding domain (RBD), which is responsible for virus binding to the cellular receptor, human angiotensin-converting enzyme-2 (hACE2), and possibly other receptors, such as heparin and the tyrosine-protein kinase receptor UFO (AXL).^10,11^ In the prefusion state, S1 subunits rest above S2 subunits, preventing conformational rearrangement of S2 subunits from a metastable prefusion conformation to a highly stable postfusion conformation.^12^ Hence, the viral S1 subunit, especially the RBD, is exposed and vulnerable to host immunity. Indeed, under the pressure of host immunity, some mutations, which have occurred in the RBD, such as N501Y, K417N, and E484K, are found in numerous variants, including B.1.1.248 and B.1.351 dominant variants.^7,8^ These mutations may play important roles in increasing viral fitness or were shown to reduce the neutralizing efficacy of RBD-specific antibodies.^13–16^ These alarming facts call for the development of broad-spectrum inhibitors targeting conserved sites in the S protein to block infection by original SARS-CoV-2 as well as its emerging variants.^17^

After receptor engagement by RBD and proteolytic cleavage activation by cellular proteases, such as TMPRSS2 and cathepsin L, a series of changes is triggered in the S2 subunit. The first involves insertion of the fusion peptide into cellular membrane, followed by HR1-trimer formation by trimeric heptad repeat 1 (HR1), which expose three hydrophobic grooves on their surface that then interact with the trimeric heptad repeat 2 (HR2) domains to form a six-helix bundle (6-HB). This 6-HB formation brings viral and cellular membranes into close proximity and is crucial for viral fusion and entry into the host cell.^9,18,19^ Hence, the HR1 region is transiently exposed, but only when the S2 subunit is transiting to a more stable postfusion conformation, effectively insulating it from host antiviral immunity. Indeed, the HR1 region is much more conserved than the RBD.^20^ Therefore, the SARS-CoV-2 HR1 region can serve as a conserved and critical target site for the development of broad-spectrum antiviral agents against SARS-CoV-2 and its variants.^21^

Previously, we have reported that OC43-HR2-derived peptide, EK1, and its lipopeptide, EK1C4, as pan-CoVs fusion inhibitors, target the HR1 regions of S proteins of multiple HCoVs and effectively inhibit their infection.^9,20^ Nevertheless, the structural and functional basis of EK1 peptides against SARS-CoV-2 remains unclear. Their broad-spectrum potency against the recently emerged SARS-CoV-2 variants, and, especially, their in vivo efficacy for COVID-19 are worthy of further study. Here, we further evaluated their anti-SARS-CoV-2 activity and found that they could potently inhibit pseudotyped and authentic SARS-CoV-2 infection in both human lung-derived and intestine-derived cell lines, Calu-3 and Caco2, respectively. More importantly, they also efficiently inhibited infections of pseudovirus carrying mutations in S proteins of recently emerged SARS-CoV-2 variants with increased transmissibility and tolerance to neutralizing antibodies used for COVID-19 therapy, and the sera from vaccinated individuals by the approved COVID-19 vaccine (BNT162b2).^16^ EK1 and EK1C4 peptides exhibited potent in vivo efficacy against SARS-CoV-2 infection on hACE2 transgenic mice, and EK1 showed ideal in vivo safety profiles, thus providing important information for prospective clinical studies of these pan-CoV fusion inhibitors in the near future. Structural study of EK1 in complex with SARS-CoV-2 HR1 not only revealed the specific mechanism of action of EK1 peptides against infection by SARS-CoV-2 and its variants, but also explained how EK1 peptides could maintain its pan-CoV inhibitory activity through adaptive side-chain conformations against HR1 motif of multiple HCoVs.

## Results

### Fusion inhibitory activity of EK1 peptides against SARS-CoV-2 infection in lung-derived and intestine-derived cell lines

**Fig. 1:**
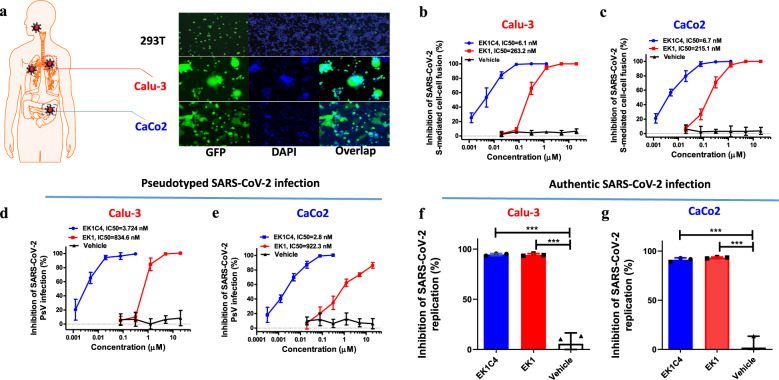
Fusion inhibitory efficacy of EK1 peptides against SARS-CoV-2 on Caco2 and Calu-3 cells. **a** The fusogenic capacity of SARS-CoV-2 S protein on, lung cell line, Calu-3 (middle) and human intestinal cell line, Caco2 (bottom), 293T cell line (upper) was used as control. **b**, **c** Fusion inhibitory activity of EK1 and EK1C4 against the SARS-CoV-2 S-mediated fusion process on Calu-3 (**b**) and Caco2 (**c**) cells. **d**, **e** Inhibitory activity of EK1 and EK1C4 against pseudotyped SARS-CoV-2 infection on Calu-3 (**d**) and Caco2 (**e**) cells. **f**, **g** Antiviral effect of EK1 (5 μM) and EK1C4 (0.3 μM) against authentic SARS-CoV-2 infection on Calu-3 (**f**) and Caco2 (**g**) cells. Viral supernatant was collected 48 h of post-infection and quantified by qRT-PCR. ***P < 0.001

Considering severe respiratory and gastrointestinal manifestations in COVID-19,^22,23^ we explored SARS-CoV-2 S-mediated fusion capacity on lung-derived and intestine-derived cell lines, Calu-3 and Caco2, respectively. Indeed, effector cells (293T/S/GFP) expressing wild-type (WT) SARS-CoV-2 S protein on the membrane surface and green fluorescent protein (GFP) in the cytoplasm could significantly fuse with both Calu-3 and Caco2 cells. As shown in Fig. 1a, after coculture for 12 h, GFP in effector cells diffused into the fused Calu-3 or Caco2 cells, and prominent syncytia were formed. These results are consistent with the tissue-susceptibility seen in COVID-19 patients.^4,5^ We then evaluated the fusion inhibitory activity of EK1 peptides in these fusion models. Both EK1 and EK1C4 potently inhibited the fusion process with Calu-3 cells with a concentration for 50% inhibition (IC_50_) of 263.2 and 6.1 nM, respectively (Fig. 1b). Similarly, EK1 and EK1C4 showed potent fusion-inhibitory activity on Caco2 cells with IC_50_ values of 215.1 and 6.7 nM, respectively (Fig. 1c). In addition, EK1 and EK1C4 effectively inhibited SARS-CoV-2 pseudovirus (PsV) infection in both Calu-3 and Caco2 cells, with IC_50_s ranging from 834.6 to 922.3 nM and 2.8 to 3.7 nM, respectively (Fig. 1d, e). Next, we evaluated these peptides for their antiviral activity against authentic SARS-CoV-2 (WT) infection. As shown in Fig. 1f, g, EK1 (5 μM) and EK1C4 (0.3 μM) could significantly inhibit authentic SARS-CoV-2 replication in both Calu-3 and Caco2 cells. Together, these results show that EK1 peptides can protect lung-ddrived and intestine-derived cell lines from SARS-CoV-2 infection suggesting that these peptides may also be effective against SARS-CoV-2 infection in lung and intestine in vivo.

### Potent inhibitory activity of EK1 peptides against SARS-CoV-2 and its variants

**Fig. 2:**
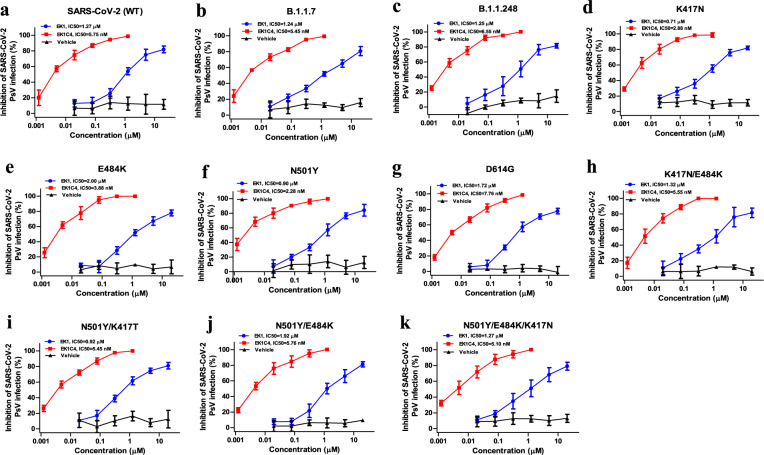
Broad-spectrum inhibitory activity of EK1-peptides against infection mediated by mutant SARS-CoV-2 S proteins. **a** Antiviral activity of EK1 and EK1C4 against wild-type SARS-CoV-2 PsV infection on Caco2 cells. **b**, **c** Inhibitory activity of EK1 and EK1C4 against infection mediated by S protein of SARS-CoV-2 B.1.1.7 (**b**) and B.1.1.248 (**c**) variants on Caco2 cells. **d**–**g** Anti-SARS-CoV-2 efficacy of EK1 and EK1C4 on PsV infection mediated by the S protein with single mutations, including K417N (**d**), E484K (**e**), N501Y (**f**), or D614G (**g**), respectively on Caco2 cells. **h-k** Antiviral efficacy of EK1 and EK1C4 against PsV infection mediated by the S protein with combinational mutations, including K417N/ E484K (**h**), N501Y/ K417N (**i**) N501Y/E484K (**j**), and N501Y/ K417N/E484K (**k**), respectively, on Caco2 cells. Experiments were repeated twice, and the data are expressed as means ± SD

We then evaluated the inhibitory activity of EK1 and EK1C4 against infection by B.1.1.7 and B.1.1.248 variants, which first emerged in the United Kingdom (UK) and Brazil, respectively. We found that both peptides effectively inhibited pseudotyped B.1.1.7 and B.1.1.248 infection with IC_50_ values of ranging from 1.24 to 1.25 μM and from 5.45 to 6.55 nM, respectively, similar to those against WT SARS-CoV-2 pseudovirus (1.27 μM and 5.75 nM, respectively) (Fig. 2a–c). Most recently, the newly emerged variants carrying crucial mutations in their S proteins, e.g., K417N, E484K, N501Y or D614G, quickly became the locally dominant variants. To explore whether EK1 peptides are antivirally active against infection mediated by spike protein carrying these mutations, we assessed their antiviral efficacy on infection by pseudoviruses (PsVs) with K417N, E484K, N501Y or D614G single mutation in their S proteins. As shown in Fig. 2d–g, EK1 and EK1C4 were effective against these PsVs with IC_50_ values ranging from 0.71 to 2.00 μM and from 2.88 to 7.66 nM, respectively. Moreover, variants with combinational mutations in S protein, including K417N/E484K, N501Y/K417N, N501Y/E484K and N501Y/K417N/E484K, were still sensitive to EK1 and EK1C4 (Fig. 2h–k) with IC_50_s ranging from 0.92 to 1.92 μM and 5.10 to 5.76 nM, respectively. Recently, some variants with mutation in HR1 region were also identified (https://www.gisaid.org/), including A924V, I931F/V, I934M/T/V, K947I/R/T, D950H/N/Y, V951L, V952A and L959V. To further evaluate the broad efficacy of EK1 peptides, we assessed their antiviral activity on 15 pseudoviurses with above single mutations in HR1 region. As shown in supplementary Fig. 1, EK1 and EK1C4 could also potently inhibit infection of those mutants, which are consistent to the results from our previous studies,^20^ confirming that SARS-CoV-2 variants with mutations in HR1 domain are still sensitive to the EK1 and EK1C4 peptides. Together, EK1 peptides exhibit broad-spectrum inhibitory activity against SARS-CoV-2 and all variants tested.

### EK1 peptides protect against SARS-CoV-2 infection in hACE2-transgenic mouse models

**Fig. 3:**
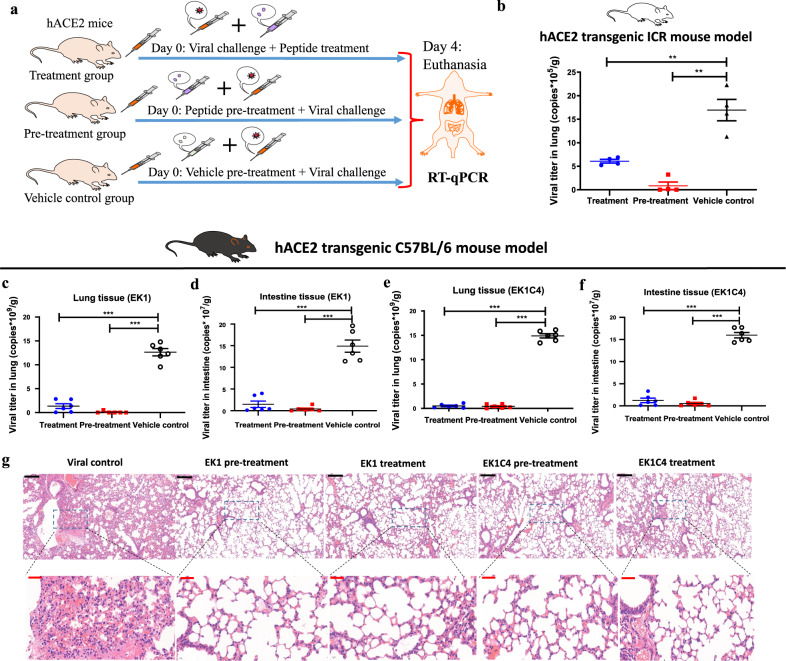
In vivo prophylactic and therapeutic efficacy of EK1 peptides against SARS-CoV-2 infection in hACE2 transgenic mouse models. **a** Schematic diagram of EK1 or EK1C4 administration and SARS-CoV-2 challenge. The mice in the treatment group were nasally administrated with a test peptide 30 min after challenge with SARS-CoV-2. The mice in the pre-treatment group and vehicle control group mice were nasally treated with a test peptide or vehicle control, respectively, 30 min before challenge with SARS-CoV-2. **b** Anti-SARS-CoV-2 efficacy of EK1 (200 μg/mouse) in hACE2 transgenic ICR mouse model. Viral titer in lung tissue of mice in each group on the 4th day of post-infection was detected. **c**, **d** Anti-SARS-CoV-2 efficacy of EK1 (200 μg/mouse) in vivo on hACE2 transgenic C57BL/6 mouse model. Viral titer in mouse lung (**c**) or intestine (**d**) of each group on the 4th day of post-infection was detected. **e**, **f** Anti-SARS-CoV-2 potency of EK1C4 (10 μg/mouse) in vivo on hACE2 transgenic C57BL/6 mouse model. Viral titer in lung (**e**) or intestine (**f**) of mice in each group on 4th day after SARS-CoV-2 challenge was detected. **P < 0.01, ***P < 0.001. **g** Histopathological examination of lung tissue of Tgtn(CAG-human ACE2-IRES-Luciferase) mice with or without administration of EK1 peptides. Lungs from hACE2 transgenic C57 mice were collected on the day 4 of post-infection. The lung tissue sections were stained with hematoxylin and eosin for histopathological examination. Scale bars in black, 200 μm (upper), scale bars in red, 40 μm (bottom)

To determine the preclinical efficacy of the pan-CoV fusion inhibitors, we used the first reported SARS-CoV-2 infection mouse model.^24^ These hACE2-transgenic ICR mice could be effectively infected by SARS-CoV-2 and showed high viral titers in lung tissue at 3–5 days of post-infection, making it possible to evaluate the prophylactic and therapeutic effects of EK1 peptide before or after viral challenge.^24^ As shown in Fig. 3a, b, the viral titers in lung tissues of mice that received prophylactic or therapeutic administration with EK1 peptide (200 μg/mouse) were significantly lower than those observed in the untreated control mice, indicating potent in vivo antiviral efficacy.

To confirm and expand the above mentioned results, we used an advanced newly developed hACE2-transgenic C57BL/6 mouse model: C57BL/6-Tgtn(CAG-human ACE2-IRES-Luciferase-WPRE-polyA)Smoc,^25^ to further evaluate the preclinical efficacy of EK1 peptides against SARS-CoV-2 infection. In this mouse model, both respiratory and gastrointestinal tissues abundantly express human ACE2,^25^ thus making it susceptible to SARS-CoV-2 infection and mimicking the clinical features in human patients. Strikingly, nasal administration of EK1 peptide (200 μg/mouse) before or after SARS-CoV-2 challenge strongly reduced viral titers in both lung and intestine, compared with the vehicle control group (Fig. 3c, d). Thus, EK1 peptide exerts prophylactic and therapeutic efficacy against SARS-CoV-2 systemic infection in vivo. Considering that EK1C4 is much more potent than EK1 in inhibiting SARS-CoV-2 infection in vitro, we proceeded to test the in vivo efficacy of EK1C4 at lower dosage (10 μg/mouse). As expected, low dose EK1C4 treatment efficiently protected against SARS-CoV-2 infection in this mouse model (Fig. 3e, f). Notably, the untreated infected mice presented typical histopathology at the 4th day of post-infection, including alveolar septa thickening and interstitial infiltrates in lung tissue, while EK1 and EK1C4 effectively attenuated these pathological changes in mice receiving prophylactic and therapeutic treatment (Fig. 3g). Altogether, our data show that EK1 peptides exhibit high in vivo protective efficacy against SARS-CoV-2, as previously reported for HCoV-OC43 and MERS-CoV infection.^20^

### Structural basis for mechanism of EK1 peptides against SARS-CoV-2 infection

**Fig. 4:**
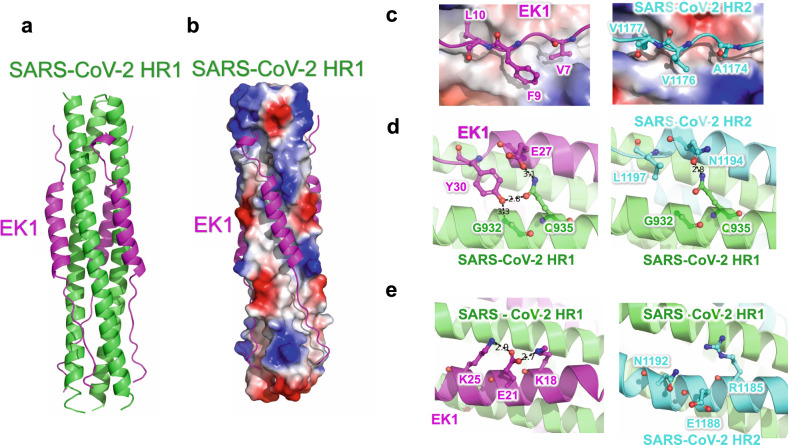
Crystal structure of 6-HB formed by SARS-CoV-2-HR1 and EK1. **a** The structure of SARS-CoV-2-HR1 and EK1 is shown in cartoon representation with HR1 colored in green and EK1 in magenta. **b** HR1-trimer of SARS-CoV-2 is shown in electrostatic surface, and EK1 is shown in cartoon representation. **c**–**e** Interactions of SARS-CoV-2 HR1 motif against EK1 peptide and SARS-CoV-2 HR2 motif. Important binding residues are shown in sticks and labeled

To investigate the inhibitory mechanism of the EK1 peptides, we crystallized EK1 in complex with the HR1 motif of the SARS-CoV-2 S2 subunit, which were tandem linked through a 6-residue linker (L6: SGGRGG). This crystal structure of HR1-L6-EK1 was solved by molecular replacement, using a SARS-CoV-HR1-L6-EK1 structure (PDB entry: 5ZVM) as reference, to a final resolution of 2.28 Å (Supplementary Table S1). In this structure, EK1 fits into the groove formed between two adjacent HR1 helices in antiparallel manner, resulting in a 6-HB structural mimic as tightly assembled as that of the viral trimer HR1-HR2 complex in post-fusion state (Fig. 4a).^9^ EK1 binds to HR1-trimer mainly through hydrophobic forces, including two flexible loop areas and a middle α-helix (Fig. 4b). The results showed that EK1 occupies the binding site of HR2 motif on the corresponding HR1-trimer, thereby blocking viral 6-HB formation and subsequently membrane fusion.

Compared with the 6-HB structure of SARS-CoV-2, EK1 showed enhanced interactions in several local regions. For example, the V1177, V1176, and A1174 residues in the SARS-CoV-2 HR2 motif bind to HR1 through moderate hydrophobic forces, which are enhanced by replacement with larger hydrophobic residues L10, F9, and V7 of EK1 at the corresponding sites, respectively (Fig. 4c). Y30 on EK1 can bind to backbone nitrogen atoms of G932, and also the side chain of Q935 on HR1 together with E27 on EK1 (Fig. 4d), while the E21 residue on EK1 could bind to both K25 and K18 residues through strong salt bridges (Fig. 4e). Such enhancement most likely improves the overall stability of 6-HB formed by HR1 and EK1, thus increasing the efficiency of EK1 in blocking binding of the viral HR2 motif to HR1.

**Fig. 5:**
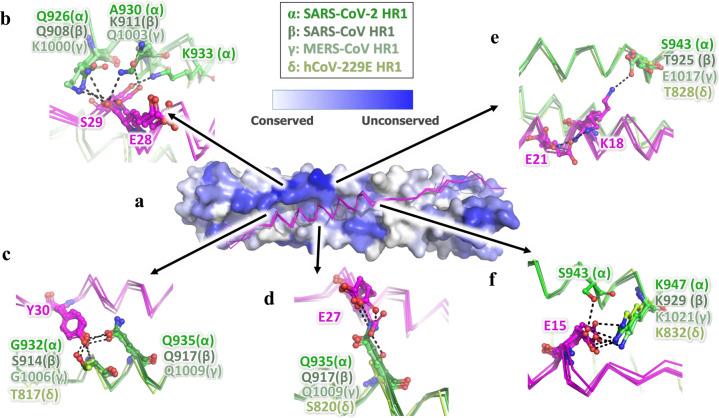
The critical residues in EK1 can flexibly interact with variable residues in HR1 region of HCoVs mainly through the hydrophilic interaction. **a** The EK1 peptides in their complex structures with SARS-CoV-2 (α, PDB entry 7C53), SARS-CoV (β, PDB entry 5ZVM), MERS-CoV (γ, PDB entry 5ZVK) and hCoV-229E (δ, PDB entry 5ZUV) are superposed together, shown as ribbon in magentas. The SARS-CoV-2 HR2 trimer is shown as surface and colored according to the residue conservations. **b**–**f** The interaction details of EK1 against HR1 in different HCoVs are shown. Important residues are shown in sticks and labeled

In structural comparisons of known HCoV-HR1-EK1 complex structures. We found that the major differences in the HR1 motif among HCoVs are on the outer surface of 6-HB located around the EK1 helix, which are mostly hydrophilic residues (Supplementary Fig. 2b and Fig. 5a). Interestingly, although some residues vary among different HCoVs, EK1 is still able to adapt to these differences and maintain strong interactions against the HR1 motif (Fig. 5 and Supplementary Fig. 3). For example, although K991 in SARS-CoV or Q1003 in MERS-CoV is replaced by A930 in SARS-CoV-2, which seems to lose its interaction with S29 of EK1, S29 turns over to bind the adjacent K933 residues in SARS-CoV-2 (Fig. 5b). Meanwhile, Q926 in SARS-CoV-2, Q908 in SARS-CoV and K1000 in MERS-CoV could all bind to the backbone carbonyl oxygen of E28 on EK1 (Fig. 5b). In a similar way, Y30 on EK1 could bind to opposite Gly in two HCoVs, Ser in another HCoV and Thr in the fourth one (Fig. 5c). E27 on EK1 could bind to Gln in three HCoVs and Ser in the fourth one in the same location (Fig. 5d).

The K18 of EK1 could bind to E1017 in HR1 of MERS-CoV, but E1017 is replaced by Ser and Thr in another 3 HCoVs, too short to bind to K18. However, in SARS-CoV-2, K18 of EK1 turns back to bind E21 on EK1 to form a new salt bridge to stabilize EK1 (Fig. 5e). E15 on EK1 could bind to the highly conserved Lys in the opposite HR1 of all 4 HCoVs, but when a new hydrophilic residue, S943, appears nearby in HR1 of SARS-CoV-2, E15 could simultaneously bind two residues of Lys and Ser on the HR1 motif (Fig. 5f). Therefore, through the variable conformations of hydrophilic residues, EK1 peptides could adapt to multiple residue differences in the HR1 motif of HCoVs, thereby maintaining high affinity to HR1 and high inhibitory activity against different HCoVs.

### Preclinical safety profiles of EK1 peptide evaluated in different animal models

**Fig. 6:**
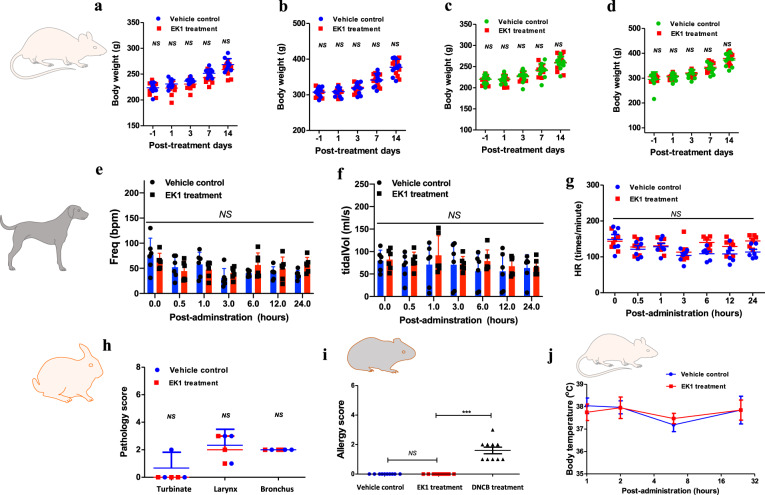
Preclinical safety of EK1 peptide in a pharmaceutical formulation. **a**–**d** In vivo safety of EK1 peptide on SD rats. The body weight of female (**a**) and male (**b**) SD rats treated by EK1 peptide (75 mg/kg) through the aerosol inhalation route. Body weight of female (**c**) and male (**d**) SD rats treated with EK1 peptide (180 mg/kg) through the tail vein injection route. **e**–**g** Effect of EK1 peptide (24 mg/kg) through the aerosol inhalation route on cardiopulmonary function in beagle dog model, including respiratory frequency (**e**), tidal volume (**f**), heart rate, HR (**g**). Those indicators were monitored by the emkaPACK4G animal physiological signal telemetry system. **h** “Nasal mucosa irritation test” of EK1 peptide (0.88 mg/kg) on a rabbit model. Pathogenic score is based on epithelial cell shedding, goblet cell hyperplasia, eosinophilic inflammatory cell invasion and mixed inflammatory cell invasion. **i** “Active skin allergy test” of EK1 peptide (4.0 mg/kg) on guinea pig model. **j** Effect of EK1 peptide (18 mg/kg) on the central nervous system. After administration of EK1 peptide through aerosol inhalation, the body temperature changes on SD rat model. ***P < 0.001; “NS” indicates no statistical difference

As the first identified peptide-based pan-CoV fusion inhibitor that started preclinical efficacy study before the COVID-19 pandemic, EK1 peptide showed effective and broad-spectrum protection in vivo against SARS-CoV-2 infection in this study as well as HCoV-OC43 and MERS-CoV infections in our previous study.^20^ Therefore, its further development towards the clinical trial is essential. We herein conducted a series of animal experiments to evaluate the systemic preclinical safety of EK1 peptide. First, we performed an in vivo toxicity test using SD rats. Given that the respiratory tract is the most important site for SARS-CoV-2 transmission and initial spread, we herein used an aerosol inhalation apparatus to intranasally administer the EK1 peptide for evaluation of its in vivo safety profile. After administration of EK1 peptide at high dosage (75 mg/kg), we observed and recorded the changes of body weight and daily behaviors. As shown in Fig. 6a, b, both female and male SD rats treated with EK1 peptide exhibited normal body-weight increase, compared with the untreated control rats. No abnormal behaviors in the EK1-treated rats were observed. Based on our previous studies,^20^ EK1 peptide could enter the blood circulation system through intranasal administration, which is beneficial to combat systemic infection by SARS-CoV-2. Here, we further assessed the in vivo safety profile of EK1 peptide in the SD rat model through tail vein injection. As shown in Fig. 6c, d, no significant difference in the body weight of EK1-treated female or male rats was observed when compared with that of rats in the untreated control group, further confirming that EK1 peptide has a good safety profile in the SD rat model.

Second, we used a Beagle dog model to evaluate the effect of EK1 peptide on cardiopulmonary function, which is an important preclinical safety indicator for clinical agents.^26^ The emkaPACK4G animal physiological signal telemetry system was used to monitor the cardiopulmonary function of Beagle dog, mainly including respiratory frequency, tidal volume, ventilation volume per minute, heart rate (HR), systolic blood pressure (SP), and diastolic blood pressure (DP). After intranasal administration of high dosage (24 mg/kg) of EK1 peptide, which is 32-fold that of the planned clinical dosage (0.75 mg/kg), respectively, we found that Freq, tidal volume, and ventilation volume per minute of the dogs in three EK1-treated groups remained within normal ranges, compared with those in the untreated control group. Thus, intranasally applied EK1 peptide at dosages as high as 24 mg/kg through aerosol inhalation administration had no significant adverse effect on normal respiratory system function in the tested animals (Fig. 6e, f and Supplementary Fig. 4a). Similarly, no statistically significant difference was seen in HR, SP, and DP of dogs between the EK1-treated groups and normal control group (Fig. 6g and Supplementary Fig. 4b, c), suggesting that EK1 peptide had no adverse effect on heart function in the dogs tested.

Third, we performed the “nasal mucosa irritation test” of EK1 peptide on a rabbit model. The pathogenic score was tabulated based on multiple histopathological examination indexes, including epithelial cell shedding, goblet cell hyperplasia, eosinophilic inflammatory cell invasion, and mixed inflammatory cell invasion, as previously described.^27^ After single intranasal administration of EK1 peptide at a dosage of 0.88 mg/kg, the respiratory tract mucosa of rabbits in both EK1-treated and non-treated groups showed no irritation (Fig. 6h). Next, we performed an “active skin allergy test” of EK1 peptide on a guinea pig model to examine whether EK1 peptide would cause an allergic reaction on the skin after repeated exposure to EK1 peptide. As shown in Fig. 6i, the administered EK1 peptide caused no skin allergy, compared with the vehicle control group, while the 2,4-dinitrochlorobenzene (DNCB)-treated group, as positive control, showed significant allergic reaction. We also assessed the effect of EK1 peptide on the central nervous system of SD rats. All EK1-treated mice showed normal body temperature (Fig. 6j) and remained within the normal range in sensory and motor function, as well as autonomous activity after intranasal administration of EK1 peptide at a dosage of 18 mg/kg.

## Discussion

Currently, SARS-CoV-2 has spread globally and caused over 180 million infections and over 3 million deaths. Although several SARS-CoV-2 vaccines have been approved for emergency or regular application, production constraints and hoarding could limit the worldwide distribution. Moreover, numerous SARS-CoV-2 variants have emerged, some of which have even shown enhanced infectivity and pathogenicity. For example, B.1.1.7, emerging in southeastern England last November, has quickly spread to more than 30 countries, becoming the dominant circulating variant in the world.^6^ Additionally, B.1.1.248, first emerged in Brazil, has shown decreased sensitivity to neutralizing antibodies in sera from vaccinated individuals.^16,28^ Therefore, the development of effective and broad-spectrum antiviral agents against SARS-CoV-2 and its variants is urgently needed. One promising class of such antivirals is the pan-CoV fusion inhibitors targeting the conserved HR1 region in the S2 subunit of CoV S protein. Our previous studies have demonstrated that the peptide-based pan-CoV fusion inhibitor EK1 and EK1C4 potently inhibit infection of divergent HCoVs by targeting their HR1 regions.^9,20^ In this study, we showed that EK1 peptides effectively blocked infection by pseudotyped SARS-CoV-2 variants B.1.1.7 and B.1.1.248 and the PsVs carrying single or multiple crucial mutations, such as N501Y, E484K, and N417N, in S proteins of the currently dominant variants. This is consistent with previous studies showing that EK1 peptides inhibit infection by multiple HCoVs, including α-HCoVs and β-HCoVs.^20^

To define the molecular mechanism underlying the broad activity of the EK1 peptides against multiple coronaviruses, such as SARS-CoV, SARS-CoV-2, MERS-CoV, and HCoV-229E, we previously crystallized EK1 in complex with HR1 peptides derived from SARS-CoV, MERS-CoV, and HCoV-229E.^20^ In this study, we further crystallized EK1 in complex with HR1 peptide derived from SARS-CoV-2 in order to study the mechanism of action of EK1 peptides against infection by SARS-CoV-2 and its variants. In each of the four known hCoV-HR1-EK1 complex structures, the backbone of EK1 exhibits high conservation with root-mean-square deviation (RMSD) less than 1 Å (Supplementary Fig. 2a), possibly because the residues around the hydrophobic grooves of HR1 motif have high homology among different HCoVs (Supplementary Fig. 2b). For example, Val7 on EK1 binds to Leu in all four structures (L959 for SARS-CoV-2, L941 for SARS-CoV, L1033 for MERS-CoV, and L844 for HCoV-229E), and L26 on EK1 binds to Ala (A930 for SARS-CoV-2, A912 for SARS-CoV, A1004 for MERS-CoV, and A815 for HCoV-229E). Moreover, although some hydrophobic residues changed among HCoVs, they still maintain strong hydrophobicity and thus keep the hydrophobic groove intact. M16 on EK1 could bind to Leu in SARS-CoV-2 (L945), SARS-CoV (L927), and HCoV-229E (L830), but also could bind to F1019 in MERS-CoV (Supplementary Fig. 3). Therefore, the hydrophobic residues on EK1, including I5, V7, F9, L10, L12, M16, L19, A22, I23, L26, I31, L33, and L36 bind to the hydrophobic residues on HR1-trimer of different coronaviruses. Considering that the hydrophobic grooves of HR1 motif are highly conserved structures and very important for coronavirus infection, EK1 peptides can thus broadly inhibit infection of HCoVs, including those that might emerge in the future.

Manifestations in COVID-19 patients mainly involve the respiratory system.^29^ Nevertheless, researchers also found susceptibility of intestinal tissue to SARS-CoV-2 infection and frequently detected SARS-CoV-2 RNA from stool samples of COVID-19 patients, indicating possible “fecal-oral” transmission of SARS-CoV-2.^30^ Consistently, we herein found SARS-CoV-2 S protein to exhibit highly fusogenic activity on Calu-3 cells derived from lung tissue and on Caco2 cells derived from intestinal tissue. Actually, human lung and intestine highly expressed TMPRSS2, just like Calu-3 and Caco2 cell lines, facilitating SARS-CoV-2 entry into host cells through the plasma membrane fusion pathway.^31–34^ Indeed, an endocytosis inhibitor, such as chloroquine, showed little effectiveness against SARS-CoV-2 infection on lung cells.^35^ On the contrary, pan-CoV fusion inhibitors, EK1 peptides, effectively block plasma membrane fusion and show potent fusion inhibitory activity on both Calu-3 and Caco2 cells. More importantly, on different mouse models, single administration of EK1 peptides significantly reduced the viral titer in mouse lung tissue, indicating that EK1 peptides possesses good preventive and therapeutic efficacy against SARS-CoV-2 infection in vivo. Particularly, in the C57BL/6-Tgtn(CAG-human ACE2-IRES-Luciferase-WPRE-polyA)Smoc mouse model, intranasally applied EK1 and EK1C4 peptides potently inhibited SARS-CoV-2 infection in both lung and intestinal tissues. They also effectively attenuated the pathological changes caused by SARS-CoV-2 infection, suggesting that both EK1 peptides are promising candidates for further development as safe and effective broad-spectrum anti-coronavirus drugs for prevention and treatment of SARS-CoV-2 infection.

In the preclinical safety study of EK1 peptide, we adopted various animal models to systemically assess multiple safety profiles, including the “in vivo toxicity test” and “central nervous system toxicity test” on an SD rat model, “cardiopulmonary function evaluation” on a beagle dog model, “nasal mucosa irritation test” on a rabbit model, and “active skin allergy test” on a guinea pig model. All results suggest that EK1 peptide has good safety profiles, i.e., lack of any significant side effects on the respiratory system, including nose, throat, bronchial tissue, and on cardiopulmonary function and the CNS, and could not raise an allergic reaction. Together, these results pave the way for subsequent clinical development of EK1 peptide as a pan-CoV fusion inhibitor against infection by SARS-CoV-2 and its variants, as well as emerging and re-emerging highly pathogenic HCoVs. The licensed peptide-based HIV-1 fusion inhibitor, T20 (enfuvirtide), has been widely used in clinic for treatment of HIV-1-infected patients who have failed to respond to other classes of anti-HIV drugs.^36^ The peptide-based pan-CoV fusion inhibitors in this study even have better potential to be used in clinic for not only treatment, but also prevention of SARS-CoV-2 infection via intranasal application. Another advantage of using EK1 peptide to treat SARS-CoV-2 infection over the application of T20 peptide to treat HIV infection is that, the anti-SARS-CoV-2 peptide has much less chance to induce drug-resistant mutations or anti-drug antibodies than the anti-HIV peptide, since the anti-SARS-CoV-2 peptide will be used in a short period of time (e.g., 2 weeks) for treatment of acute SARS-CoV-2 infection, while the anti-HIV peptide has to be used for a long period of time (e.g., more than 1 year).

## Materials and methods

### Cells, viruses, and peptides

Calu-3, Caco2, and 293T cells were obtained from ATCC (Manassas, VA, USA). Huh-7 cells was from the Chinese Academy of Science Cell Bank (Shanghai, China). Cells were cultured in Dulbecco’s Modified Eagle’s Medium (DMEM) or with 10% fetal bovine serum (FBS). Patient-derived SARS-CoV-2 (nCoV-SH01, GenBank number: MT121215.1) was isolated by Fudan University. The sequences of EK1 and EK1C4, as previously described,^9^ were synthesized by Chao Wang (Institute of Pharmacology and Toxicology, Beijing).

### Expression and purification of fusion protein of HR1(SARS-CoV-2)-L6-EK1

The coding sequences of SARS-CoV-2 S2 subunit HR1 motif (residues 910–974) and EK1 peptide (SLDQINVTFLDLEYEMKKLEEAIKKLEESYIDLKEL) were tandem linked though a 6-residue linker (L6: SGGRGG). The resulting sequences encoding the fused HR1-L6-EK1 protein were then cloned into a modified pET-28a vector containing a His_6_-SUMO tag upstream of the multiple cloning sites. The recombinant construct was expressed in *Escherichia coli* BL21 (DE3). Cells were grown in lysogeny broth (LB) media supplemented with 50 μg/mL kanamycin at 37 °C and were induced with 0.2 mM IPTG for 16 h at 20 °C overnight. Cells were harvested by centrifugation at 5000 × *g* for 6 min at 4 °C and were lysed by high-pressure homogenizer twice after resuspension in buffer containing 25 mM Tris–HCl, pH 8.0, and 200 mM NaCl. The fusion proteins were isolated by Ni-affinity chromatography, and the SUMO tag was removed by Ulp1 enzyme (1:200 w/w) cleavage. HR1-L6-EK1 protein was concentrated and gel-filtered on a 10/300 Superdex 75 (GE Healthcare) column. Peak fractions containing HR1-L6-EK1 trimer were pooled and concentrated to 20 mg/mL through centrifugation (EMD Millipore).

### Crystallization and structure determination

Crystals were obtained at 16 °C for 5 days using the hanging drop vapor diffusion method by mixing equal volume of protein solution (HR1-L6-EK1, 20 mg/mL) and reservoir solution (30% MPD, 0.1 M Na Acetate, pH 5.0, 0.02 M Calcium Chloride). Then crystals were flash-frozen and transferred to liquid nitrogen for data collection. On the in-house (Institute of Biophysics, Chinese Academy of Sciences) X-ray source (MicroMax 007 generator (Rigaku, Japan)) combined with Varimax HR optics (Rigaku, Japan), HR1-L6-EK1 crystals at 100 K were diffracted to 2.28-Å resolution at a wavelength of 1.5418 Å. A native set of X-ray diffraction data was collected with the R-AXIS IV ++ detector (Rigaku, Japan) with an exposure time of 3 min per image and was indexed and processed using XDS.^37^ The space group of the collected dataset was C 2 2 21. Molecular replacement was performed with PHENIX.phaser^38^ to solve the phasing problem, using crystal structure of SARS-CoV HR1 in complex with EK1 (PDB entry: 5ZVM) as a search model. The final model was manually adjusted in COOT^39^ and refined with Phenix.refine.^40^ Data collection statistics and refinement statistics are given in Table S1. Coordinates were deposited in the RCSB Protein Data Bank (PDB entry: 7C53).

### Inhibition of pseudotyped virus infection

Pseudovirus bearing wild or mutant CoVs S protein and a defective HIV-1/VSV genome by which luciferase reporter was produced, as previously described,^9^ were used to infect target Caco2 cells (HIV-1 backbone-based PsV) and Calu-3 cells (VsV backbone-based PsV) in the presence or absence of each peptide at indicated concentrations, respectively. After 12 h, culture medium was refreshed and cultured an additional 48 h. Then, cells were washed with PBS, lysed with lysis reagent (Promega), and relative light units (RLU) detected by using Luciferase Assay Kits.

### Establishment of multiple HCoVs S-mediated cell–cell fusion

As described elsewhere,^9^ effector cells (293T/S/GFP) were produced by transfecting the plasmid pAAV-IRES-S-EGFP into 293T, which encoded SARS-CoV-2 S protein and EGFP. Caco2 and Calu-3 cells were used as target cells. Effector cells were co-cultured with target cells in the presence or absence of peptides at the indicated concentrations for fusion. Finally, the fused and unfused cells were counted under an inverted fluorescence microscope (Nikon Eclipse Ti-S).

### Inhibition of SARS-CoV-2 replication

A live SARS-CoV-2 experiment was performed in a biosafety level 3 facility at Fudan University. Briefly, 50 μL of SARS-CoV-2 (750 pfu/mL) were premixed with 50 μL of culture media containing EK1 or EK1C4 for 30 min. Then, the mixture was added to Calu-3 or Caco-2 cells. After culture for 48 h, the supernatant was harvested to evaluate the viral RNA levels by qRT-PCR.^41^

### RT-qPCR

Viral RNA was extracted from supernatants using the EasyPureViral DNA and RNA Kit (TransGen, China) and then measured using a One-Step PrimeScript RT-PCR Kit (Takara, Japan). The sequences of probe and primers are as follows:

Probe: 5′-FAM-CCGTCTGCG GTATGTGGAAAGGTTATGG-BHQ1-3′

SARS-CoV-2-ORF1ab-F: CCCTGTGGGTTTTACACTTAA;

SARS-CoV-2-ORF1ab-R: ACGATTGTGCATCAGCTGA.

### Mouse infection studies

All in vivo studies were performed in accordance with the animal experiment protocol approved by the Animal Experiment Committee of the School of Basic Medical Sciences, Fudan University (20210302-083). Male hACE2 transgenic ICR mice were purchased from the Institute of Laboratory Animal Science, Peking Union Medical College, China. Mice were administered intranasally with EK1 (200 μg/mouse) 30 min before or after viral challenge with 10^7^ pfu SARS-CoV-2 intranasally. On the 4th day of post-infection, mice were euthanized, and the lungs were collected for viral RNA level examination.

Female hACE2 transgenic mice, C57BL/6-Tgtn(CAG-human ACE2-IRES-Luciferase-WPRE-polyA)Smoc, were from Shanghai Model Organisms, China. Mice were administered intranasally with EK1 (200 μg/mouse) or EK1C4 (10 μg/mouse) 30 min before or after viral challenge with 30,000 pfu SARS-CoV-2. On the 4th day of post-infection, mice were euthanized, and lung and intestinal tissues were collected to examine viral RNA level and pathological changes.

### Evaluation of preclinical safety of EK1

According to good laboratory practice (GLP) standard,^42^^,43^ the preclinical safety of EK1 was performed as follows.

Acute in vivo toxicity test of EK1 on SD rats was performed as previously described.^42^ Briefly, EK1 was administered through two routes, including tail vein injection (180 mg/kg) or aerosol inhalation (75 mg/kg). For each route, SD rats were divided in four groups (*n* = 10); EK1-treated female group, control female group, EK1-treated male group, and control male group. After EK1 administration, behavior, appearance and body weight changes were observed and recorded.

The effect of EK1 on cardiopulmonary function was evaluated on a beagle dog model as previously described.^43^ After intranasal administration of EK1 (24 mg/kg), the cardiopulmonary function of beagle dogs was monitored by using the embaPACK4G animal physiological signal telemetry system, mainly including respiratory frequency, tidal volume, minute ventilation volume, heart rate (HR), systolic blood pressure (SP), and diastolic blood pressure (DP).

The “nasal mucosa irritation test” of EK1 was performed on a rabbit model, as previously described.^27^ Briefly, rabbits were administered with EK1 (0.88 mg/kg) through the aerosol inhalation route. Rabbits were euthanized, and turbinate, larynx and bronchus tissues were collected for histopathological examination. Pathological degree was scored, and scores 0, 1, 2, 3 represent normal, mild, moderate, and severe for each histopathological index, including epithelial cell shedding, goblet cell hyperplasia, eosinophilic inflammatory cell invasion, and mixed inflammatory cell invasion. The final pathological score was the cumulative score.

Skin allergy test of EK1 was performed on a guinea pig model as described.^44^ Briefly, forty guinea pigs were randomly divided into vehicle control group, DNCB-treatment group, and EK1-treated group (4.0 mg/kg). Sensitization was carried out on the depilated area of left back three times on the 0th, 7th, and 14th day. Then, guinea pigs were challenged on the 14th day after the last sensitization. Skin erythema, edema, and systemic allergic reaction were observed 1 h after challenge. Allergenic reaction indexes, including erythema and swelling, were scored as follows: 0, no visible change; 1, mild erythema or swelling; 2, moderate erythema or swelling; 3, intense erythema or swelling. The final allergy score was the cumulative score.

Effect of EK1 on the central nervous system was assessed in the SD rat model as previously reported^42^. Twenty SD rats were divided into two groups (*n* = 10) and were administered by control (0.9% NaCl) or EK1 (18 mg/kg), respectively, through the aerosol inhalation route. Sensory function, motor function, and autonomous activity of SD rats were observed at 1, 2, 6, and 24 h after administration; meantime and body temperature were measured and recorded.

### Statistical analyses

Analyses of independent data were performed by using Student’s unpaired two-tailed *t*-test and analysis of variance (ANOVA) test. Statistical analyses were carried out using GraphPad Prism 5.0. *P* values less than 0.05 were significant; *P < 0.05; **P < 0.01; ***P < 0.001; ****P < 0.0001. The concentration for half inhibition (IC_50_) was calculated by using the CalcuSyn software.^45^

## Supplementary information

SUPPLEMENTAL MATERIAL

## Data Availability

The data sets of the study are available from the corresponding authors upon reasonable request.
