# Supplementary material for: Structural and functional basis for pan-CoV fusion inhibitors against SARS-CoV-2 and its variants with preclinical evaluation
Source: Signal Transduct Target Ther. 2021 Jul 29;6:288. doi: 10.1038/s41392-021-00712-2 (PMC8320318; doi:10.1038/s41392-021-00712-2)
Supplement: Supplementary file 1 — SUPPLEMENTAL MATERIAL [file 41392_2021_712_MOESM1_ESM.docx]

Supplementary Materials for

“Structural and functional basis for pan-CoV fusion inhibitors against SARS-CoV-2 and its variants with preclinical evaluation”

Shuai Xia^1^*, Qiaoshuai Lan^1^*, Yun Zhu^2^*, Chao Wang^3^*, Wei Xu^1^*, Yutang Li^1^, Lijue Wang^1^, Fanke Jiao^1^, Jie Zhou^1^, Chen Hua^1^, Qian Wang^1^, Xia Cai^1^, Yang Wu^1^, Jie Gao^4^, Huan Liu^4^, Ge Sun^4^, Jan Münch^5^, Frank Kirchhoff ^5^, Zhenghong Yuan^1^, Youhua Xie^1^, Fei Sun^2, 6^†, Shibo Jiang^1^†, Lu Lu^1^†

^1^Key Laboratory of Medical Molecular Virology (MOE/NHC/CAMS), School of Basic Medical Sciences and Biosafety Level 3 Laboratory, Shanghai Institute of Infectious Disease and Biosecurity, Fudan University, Shanghai 200032, China.

^2^ National Key Laboratory of Biomacromolecules, CAS Center for Excellence in Biomacromolecules, Institute of Biophysics, Chinese Academy of Sciences, Beijing 100101, China.

^3^ State Key Laboratory of Toxicology and Medical Countermeasures, Beijing Institute of Pharmacology and Toxicology, Beijing, 100850, China.

^4^ China Institute for Radiation Protection, Taiyuan, Shanxi Province, 030006, China.

^5^ Institute of Molecular Virology, Ulm University Medical Center, 89081 Ulm, Germany.

^6^ Bioland Laboratory (Guangzhou Regenerative Medicine and Health Guangdong Laboratory), Guangzhou 510005, Guangdong Province, China

*These authors contributed equally to this work. These authors are co-senior authors: Zhenghong Yuan, Youhua Xie, Fei Sun, Shibo Jiang, Lu Lu

†Corresponding author. Email: lul@fudan.edu.cn (L.L.); shibojiang@fudan.edu.cn (S.J.); feisun@ibp.ac.cn (F.S.).

**This file includes:**

Figures S1 to S4

Tables S1


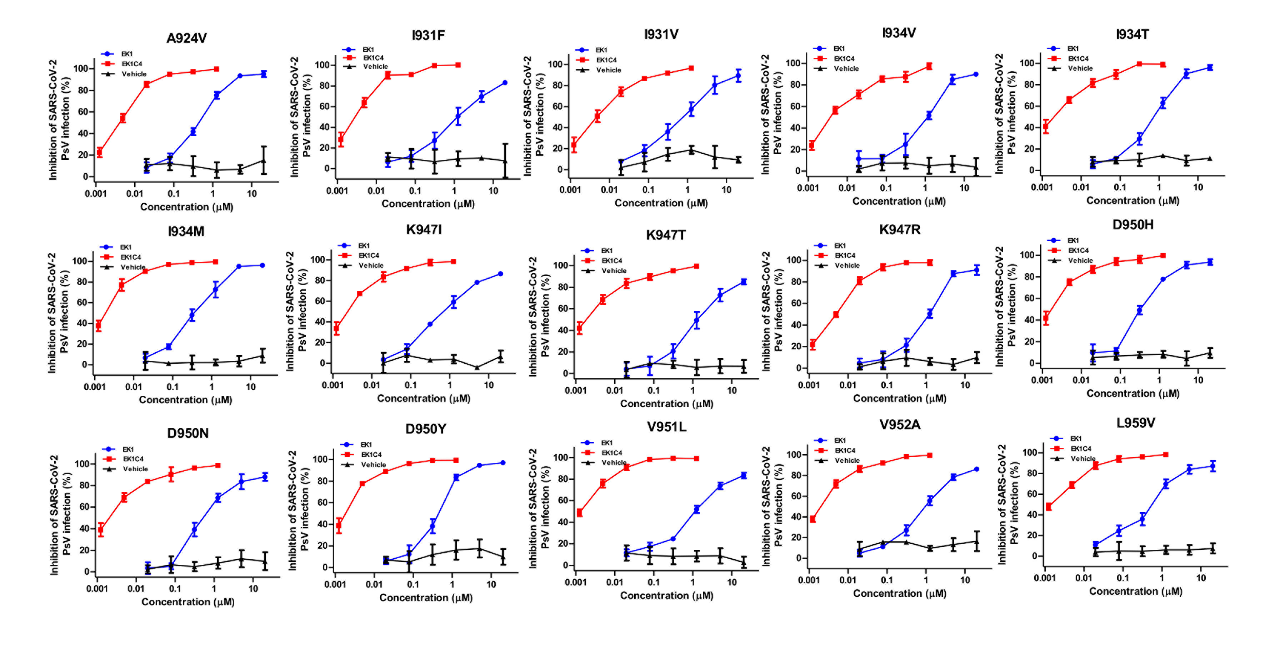


**Figure S1. Broad-spectrum inhibitory activity of EK1-peptides against infection of SARS-CoV-2 pseudoviruses with single mutation in HR1 of their S proteins.**

**
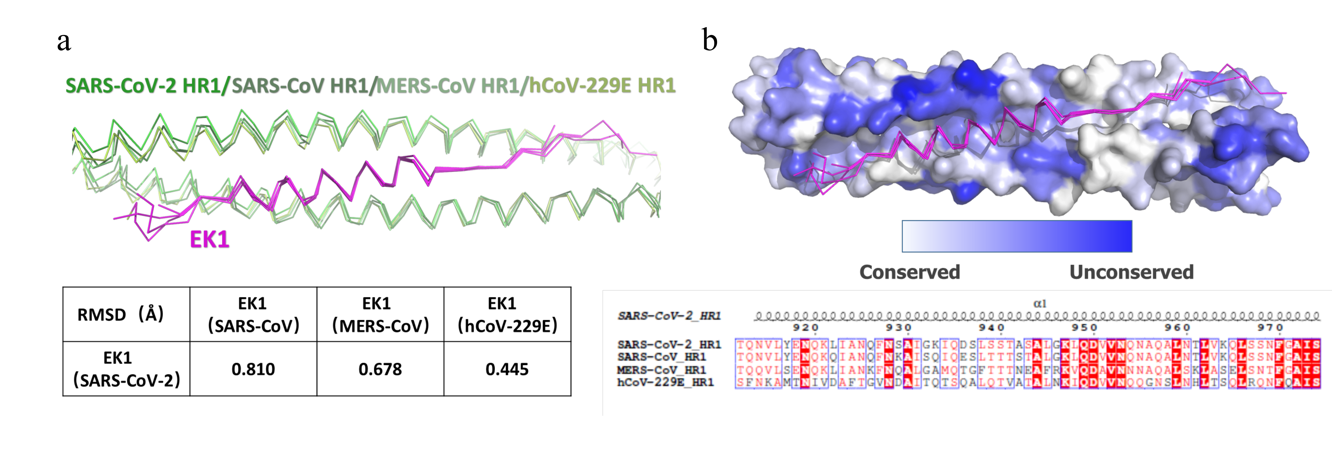
**

**Figure S2. EK1 peptide binds to HCoVs’ HR1 motif in a conserved manner. a** The superposition of EK1 structure against HR1 motif of SARS-CoV-2, SARS-CoV (PDB entry 5ZVM), MERS-CoV (PDB entry 5ZVK) and hCoV-229E (PDB entry 5ZUV). RMSD among four structures is indicated. **b** Conservation of HR1 residues among four HCoVs is shown as heat map from white color (highly conserved) to blue color (highly unconserved).


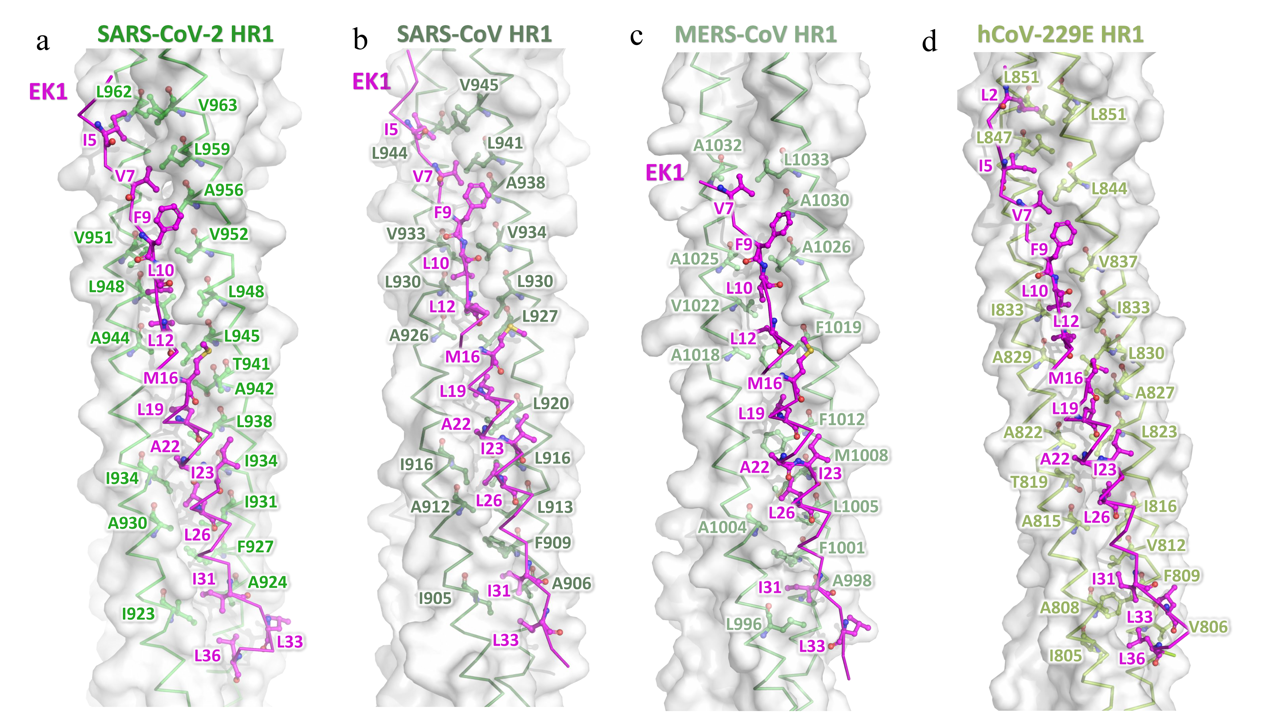


**Figure S3. Hydrophobic interactions between EK1 and four HCoVs.** **a** SARS-CoV-2 HR1 and EK1. **b** SARS-CoV HR1 and EK1. **c** MERS-CoV HR1 and EK1. **d** HCoV-229E HR1 and EK1. Important residues are shown in sticks and labeled.

**

**

**Figure S4. Effect of EK1 on cardiopulmonary function of Beagle dog model.** Effect of EK1 (24 mg/kg) through aerosol inhalation administration on cardiopulmonary function of Beagle dog model, including ventilation volume per minute (**a**), systolic blood pressure, SP (**b**), and diastolic blood pressure, DP (**c**). As compared with the vehicle control, “*NS*” indicates no statistical difference.

**Table S1. Data collection and refinement statistics**

|  | **SARS-CoV-2-HR1 / EK1**  **PDB entry 7C53** |
| --- | --- |
| **Data collection** |  |
| Space group | C 2 2 21 |
| Cell dimensions |  |
| a, b, c (Å) | 133.8, 138.1, 71.4 |
| α, β, γ (°) | 90, 90, 90 |
| Wavelength (Å) | 1.5418 |
| Resolution (Å) | 43.52 -2.28 (2.36 - 2.28) ^†^ |
| *R*_merge_ | 0.17 (1.50) |
| Mean I/σ(I) | 15.5 (2.1) |
| Completeness (%) | 99.9 (99.5) |
| Redundancy | 14.4 (13.8) |
| **Refinement** |  |
| Resolution (Å) | 43.54 -2.28 |
| No. of reflections | 30683 |
| Reflections in test set | 1491 |
| *R*_work_/R_free_ | 0.196/0.239 |
| No. of atoms |  |
| Protein | 4346 |
| Water & Ligands | 158 |
| r.m.s. deviations |  |
| Bond lengths (Å) | 0.007 |
| Bond angles (°) | 1.02 |
| Ramachandran Outliers(%) | 0 |
| Average *B*-factor (Å^2^) | 46.78 |

^†^Highest resolution shell is shown in parenthesis.
